# Recurrent chest pain as a rare presentation of extra‐pelvic endometriosis

**DOI:** 10.1002/ccr3.4413

**Published:** 2021-07-09

**Authors:** Mousa Hussein, Mutaz Albakri, Shanima Ismail, Abbas Alabbas, Samir Al Hyassat

**Affiliations:** ^1^ Pulmonary Department Hamad Medical Corporation Doha Qatar; ^2^ Laboratory Medicine & Pathology Department Hamad Medical Corporation Doha Qatar

**Keywords:** bloody effusion, effusion, endometriosis, pleural disease, thoracoscopy

## Abstract

Periodic chest pain, with bloody pleural effusion should raise the suspicion of pleural endometriosis as a well‐known, but a rare condition in clinical practice.

## Graphical abstract

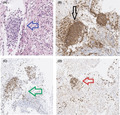

## 1 CLINICAL PRESENTATION

**FIGURE 1:**
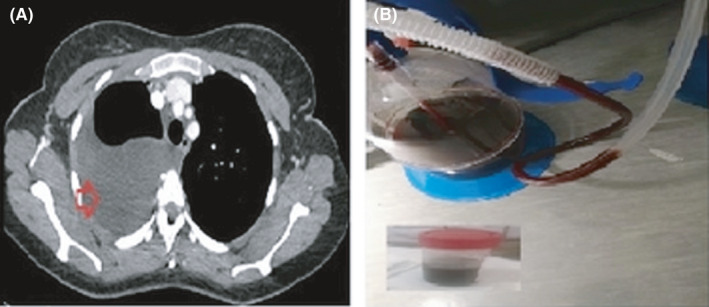
A, CT chest showing large right‐sided free‐floating effusion (red arrow). B, Chest tube with dark‐brown (Chocolate) appearance of the pleural fluid

**FIGURE 2:**
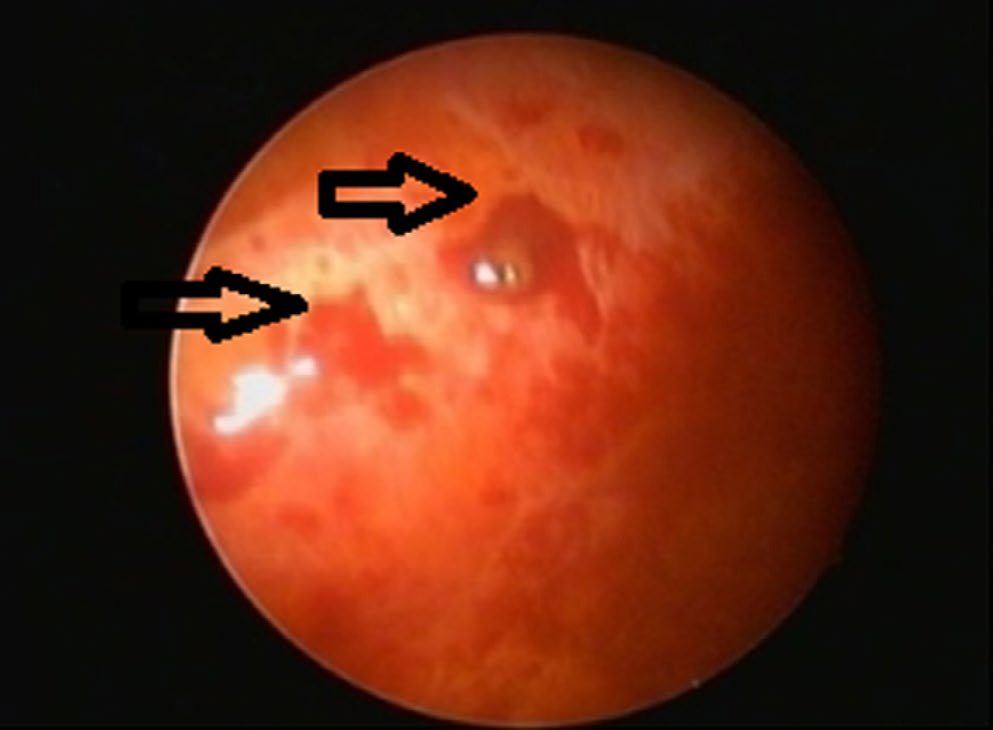
Image taken during medical thoracoscopy, showing brown pigment area of the parietal pleura

**FIGURE 3:**
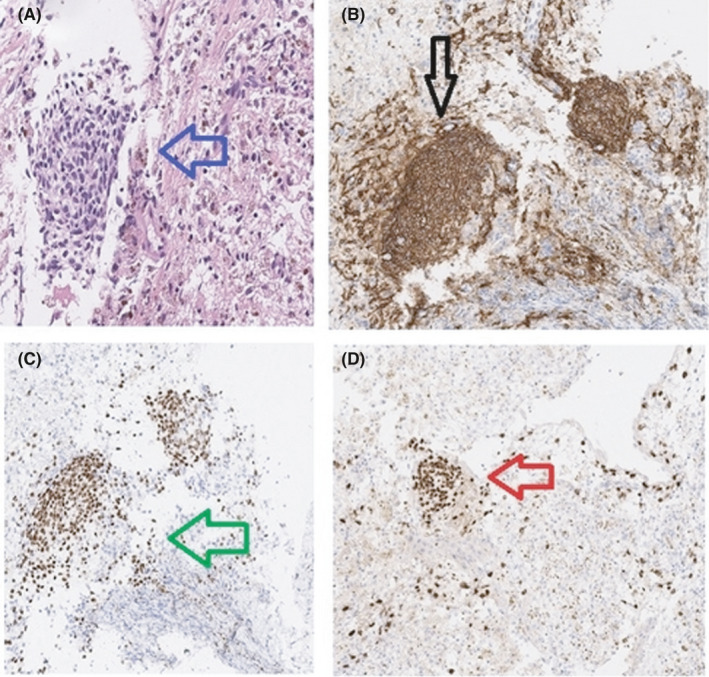
Histopathology slides A, groups of endometrial stroma‐like elements (blue arrow). The endometrial stromal cells are strongly positive for CD10 (black arrow), B, estrogen (green arrow), C, and progesterone (red arrow; D)

A 34‐year‐old woman presented with several months' history of right‐sided chest pain, that occurred at the same time as her menses every month. She had no cough, sputum production, fever, or weight loss. She is a nonsmoker, no significant past medical history, and no similar condition in her family. The physical examination was remarkable for right‐sided pleural effusion, otherwise no significant findings. Laboratory studies were notable for a hemoglobin level of 10.7 g per deciliter, normal inflammatory markers, serum protein of 79 g per deciliter, lactate dehydrogenase of 240 units per liter. Findings on X‐ray and computed tomography of the chest included large right‐sided free‐floating effusion with normal lung parenchyma (Figure 1A). The pleural fluid was dark‐brown (Chocolate effusion; Figure 1B), with red blood cells of 23 375 cells per microliter, and fluid to serum hematocrit ratio of 0.63. Fluid Lactate dehydrogenase of 142 units per liter, protein of 51 grams per deciliter, and PH of 7.45. Gram stain, culture, and tuberculosis workup from the fluid were negative. She underwent medical thoracoscopy which showed a brown‐pigmented implant (Figure 2). The pleural biopsies revealed groups of endometrial stroma‐like elements that were positive for CD10, estrogen, and progesterone (Figure 3). The periodic chest pain, with the dark‐brown appearance of the pleural fluid resulting from invading of the pleural surfaces with endometrial cells that become active during a menstrual period. The patient was referred to a thoracic surgeon and gynecologist for definitive management.

## CONFLICT OF INTEREST

None declared.

## AUTHOR CONTRIBUTIONS

MH: served as corresponding author and involved in manuscript review, writing and submission. MA: served as co‐author and involved in manuscript review, and finalization. SI, SA, and AA: involved in manuscript review

## CONSENT FOR PUBLICATION

This case report does not contain any personal identifier of the patient, for example, name and photograph. It only includes radiological and pathological imaging. A written patient informed consent was signed by the patient.

